# High Serum IgG4 Concentrations in Patients with Hashimoto's Thyroiditis

**DOI:** 10.1155/2015/706843

**Published:** 2015-02-16

**Authors:** Anna Popławska-Kita, Maria Kościuszko-Zdrodowska, Katarzyna Siewko, Beata Telejko, Justyna Hryniewicka, Robert Milewski, Saeid Soleman Abdelrazek, Małgorzata Szelachowska, Maria Górska

**Affiliations:** ^1^Department of Endocrinology, Diabetology and Internal Medicine, Medical University of Bialystok, Sklodowskiej-Curie 24A, 15-276 Bialystok, Poland; ^2^Department of Statistics and Medical Informatics, Medical University of Bialystok, 15-276 Bialystok, Poland; ^3^Department of Nuclear Medicine, Medical University of Bialystok, 15-276 Bialystok, Poland

## Abstract

*Purpose*. Since recent reports suggest that Hashimoto thyroiditis (HT) may be associated with IgG4-related disease, we aimed to find out whether the measurement of serum IgG4 allows for the identification of distinct types of HT, with different clinical, sonographic, and serologic characteristics. *Methods*. The group studied consisted of 53 patients with HT and 28 healthy individuals who underwent thyroid ultrasonography and body composition analysis. Serum concentrations of IgG4, TSH, anti-peroxidase antibodies (TPOAb), anti-TSH receptor antibodies, TNF-*α*, TGF-*β*1, Fas Ligand, TRAIL, and chemokines (CXCL9, CXCL11, and CXCL10) were measured by ELISA or radioimmunoassay. *Results*. The group with IgG4 level >135 IU/ml accounted for 32.5% of the patients. The signs of fibrosis were present in 27.0% of the high-IgG4 patients and in 9.1% of the normal-IgG4 group. The patients with elevated IgG4 required higher doses of L-thyroxine and had significantly lower level of TPOAb (*P*=0.02) than the non-IgG4-HT individuals and higher TNF-*α* level in comparison with the controls (*P*=0.01). *Conclusions*. Our results suggest that the measurement of serum IgG4 allows for an identification of patients with more rapid progression of HT, requiring higher doses of L-thyroxine. Low TPOAb level and the absence of coexisting autoimmune diseases may suggest distinct pathomechanism of this type of thyroiditis.

## 1. Introduction

Hashimoto's thyroiditis (HT, synonyms: chronic lymphocytic thyroiditis, chronic autoimmune thyroiditis)—an autoimmune disease described for the first time more than a hundred years ago [1, 2]—is regarded to be the most frequent cause of noniatrogenic hypothyroidism in all age groups. According to epidemiological data, the incidence of the disease averages from 30 to 150 cases, but some countries report as many as 500 new cases per 100 000 people every year [3–6]. The incidence of subclinical hypothyroidism is estimated to be 10–15%, yet overt hypothyroidism caused by HT affects approximately 0.1–2% of the population [5–7]. Despite a great progress made in identifying factors responsible for the development of autoimmune inflammation, the pathogenesis of HT still remains unclear. However, recent reports strongly suggest that at least some cases of HT may be closely associated with IgG4-related disease (IgG4-RD). The IgG4-related disease is a new disease entity first proposed in relation to autoimmune pancreatitis by Hamano et al. in 2001 [7]. Since then, IgG4-related lesions have been reported in various organs, including retroperitoneal fibrosis [8], sclerosing cholangitis [9], hepatic inflammatory pseudotumor [10], lymphadenopathy [11, 12], lymphoid interstitial pneumonia [13], inflammatory pseudotumor of the lung [14], orbital pseudotumor [15], sclerosing sialoadenitis (Kuttner's tumour) [16], tubulointerstitial nephritis [17], inflammatory aortic aneurysm [18], pachymeningitis [19], and dacryoadenitis (Mikulicz's disease) [20]. Clinically, IgG4-RD is characterized by high serum immunoglobulin class G4 (IgG4) levels and alleviation of symptoms after steroid therapy. Furthermore, irrespective of the organs affected, IgG4-RDs share similar pathologic features, including lymphoplasmacytic infiltration, fibrosis, obliterative phlebitis, and increased numbers of IgG4-positive plasma cells [12, 21–29]. In 2009, a group of Japanese researchers suggested that on the basis of immunostaining for IgG4, HT can be divided into two groups, which were proposed as IgG4 thyroiditis and non-IgG4 thyroiditis [30]. Histologically, IgG4 thyroiditis showed pathologic features similar to other forms of IgG4-RD, like stromal fibrosis and lymphoplasmacytic infiltration. Moreover, although the division into the two aforementioned groups was made predominantly on the basis of immunohistochemical findings, IgG4 thyroiditis was also characterized by distinct clinical, serological, and sonographic features. What is more, since increased IgG4 concentration (>134 mg/dL) was observed in the majority (70–90%) of IgG4-RD patients [30], the measurement of serum IgG4 level was proposed as a useful method of distinguishing IgG4 thyroiditis from non-IgG4 thyroiditis.

Therefore, in the present study, we aimed to find out whether the measurement of serum IgG4 allows for an identification of distinct types of HT, with different clinical and serologic characteristics. To test this hypothesis, we compared thyroid function, ultrasonographic findings, and the levels of anti-thyroid antibodies, proinflammatory cytokines, and apoptotic markers in patients with HT and low/normal and elevated levels of IgG4.

## 2. Subjects and Methods

### 2.1. Study Group

The study comprised 81 subjects, including 53 patients with diagnosed HT (mean age 44.6 ± 15.3 years, F/M 90.6%/9.4%) and 28 healthy individuals (mean age 40.8 ± 15.6 years, F/M 89.3%/10.7%), who had never been treated for any autoimmune or malignant diseases, matched for age and gender. None of the participants was suspected/diagnosed of having any IgG4-RD (except IgG4-HT). All patients and controls gave their informed consent to participate in the study before enrolment. The protocol was approved by the local ethics committee (Medical University of Bialystok, Poland).

All the patients underwent thyroid ultrasonography to establish thyroid volume, echogenicity, vascularity (with Power Doppler method), signs of fibrosis, and calcification.

A body composition analysis was performed using the medical body analyzer INBODY 220 (*Biospace, Korea*), which allows measurement of body mass, total body water (TBW), fat and free fat mass, skeletal muscle mass (SMM), BMI, percentage of body fat (PBF), and basic metabolic rate (BMR). The reliability of an estimated BMR was confirmed by the calorimetric method performed in 5 patients with HT. Differences between these results were smaller than 5%. The waist to hip ratio (WHR) was calculated as waist circumference in centimeters divided by hip circumference in centimeters.

### 2.2. Analytical Methods

Blood samples were collected from the antecubital vein between 7:30 and 8:30 am, after an overnight fast, in order to avoid diurnal variations. Serum TSH concentration was measured using an enzyme-linked immunoassay (DiaSource, Louvain-la-Neuve, Belgium). Anti-peroxidase antibodies (TPOAb) and tumor necrosis factor-alpha (TNF-*α*) levels were also determined by commercial immunoassays (Euroimmun, Lubeck, Germany, and R&D Systems, Minneapolis, USA). The concentration of anti-TSH receptor antibodies (TRAb) was measured by a commercial radioimmunoassay (TRAK HUMAN, B-R-A-H-M-S Berlin, Germany). IgG4 was estimated using Human IgG4 Platinum ELISA (eBioscience, Vienna, Austria). The following chemokines and apoptotic markers were also measured: Human Fas Ligand/TNFSF6, Human TRAIL/TNFSF10, Human TGF-*β*1, and chemokines CXCL9/I-TAC, CXCL10/IP-10, and CXCL11/I-TAC (Human Quantikine ELISA R&D Systems, Minneapolis, USA).

### 2.3. Statistical Analysis

STATISTICA 10.0 for Windows (StatSoft. Inc., USA) and IBM SPSS Statistics 21.0 (Predictive Solutions, USA) Software were used for the statistical analysis. Prior to the analysis, data were tested for normality of distribution using Kołmogorow-Smirnow test with Lilliefors correction and Shapiro-Wilk test. Differences between the groups were compared by Mann-Whitney* U* test. *P* value lower than 0.05 was considered statistically significant.

## 3. Results

The HT group was divided into IgG4-HT (IgG4 > 135 IU/mL) and non-IgG4-HT (IgG4 < 135 IU/mL), depending on the patient's IgG4 level. The group with IgG4 >135 IU/mL accounted for 32.5% of all HT patients. The percentage of male patients was 7.5% in the non-IgG4-HT and 18.2% in the group with a high level of IgG4. Mean HT duration was 4.9 ± 4.6 years in non-IgG4-HT group and 2.5 ± 1.96 in IgG4-HT group (*P* < 0.001). Furthermore, the IgG4-HT patients required higher L-thyroxine dose in order to reach euthyreosis in comparison with the non-IgG4-HT group (Table 1); however, the difference was not significant. The medical history of IgG4-HT patients revealed no presence of autoimmune disorders, whereas 15% of patients from the non-IgG4-HT group had other autoimmune diseases, such as vitiligo (7.5%), type 1 diabetes, and celiac disease (2.5%).

Ultrasound examination revealed a frequent occurrence of decreased echogenicity of the thyroid in both non-IgG4-HT and IgG4-HT patients (86.1% versus 81.8%), as well as a comparable percentage of thyroid calcifications (9.1% versus 13.9%). The signs of fibrosis were present in 27.0% of IgG4-HT patients in comparison with 9.1% of the non-IgG4-HT group, but the difference was not significant. The non-IgG4-HT patients had a lower number of thyroid nodules compared with IgG4-HT patients (*P* = 0.02) (Table 1), and 66.7% of all nodules were hypoechogenic. As expected, the HT patients (both IgG4-HT and non-IgG4-HT) had a significantly smaller thyroid volume (*P* = 0.001) in comparison with the control group (*P* = 0.001), whereas TSH values did not differ markedly between the groups studied (Table 1).

The analysis of anti-thyroid antibodies revealed that the patients with elevated IgG4 had significantly lower level of TPOAb (*P* = 0.02) than the non-IgG4-HT individuals (*P* = 0.02), whereas TRAb concentrations were similar in both groups with HT (Table 1). The concentrations of both anti-thyroid antibodies in the IgG4-HT group was markedly higher in comparison with the controls (*P* = 0.0001, Table 1).

As shown in Table 2, body composition did not differ significantly between the IgG4-HT and non-IgG4-HT subgroups; however the patients with elevated IgG4 had significantly higher body mass (*P* = 0.008), BMI (*P* = 0.02), WHR (*P* = 0.01), and fat mass (*P* = 0.02) in comparison with the healthy subjects.

As shown in Table 3, the level of IgG4 was higher in the IgG4-HT than in the non-IgG4-HT (by definition, *P* = 0.001) and in the control group; however, the difference between the IgG4-HT and healthy individuals was not significant. Serum TNF-*α* level was markedly higher in the IgG4-HT in comparison with the controls (*P* = 0.01). There was also a trend towards higher TGF-*β*1 concentration in the IgG4-HT, as compared with the non-IgG4-HT group (*P* = 0.05), whereas the levels of other chemokines and apoptotic markers (Fas Ligand and TRAIL) were similar in all groups studied (Table 3).

## 4. Discussion

Despite the growing number of studies on various forms of IgG4-related diseases, clinical reports focused on IgG4-thyroiditis seem scarce and nonconclusive [22–26]. However, it has been suggested that IgG4-HT is characterized by distinct clinical, serological, and sonographic signs distinguishing it from non-IgG4-HT. In particular, Kakudo et al. [31] showed that clinically IgG4-HT was associated with younger age, lower female-male ratio, higher levels of thyroid autoantibodies, diffuse low echogenicity, and more subclinical hypothyroidism. Histopathologically, IgG4 thyroiditis showed a higher grade of stromal fibrosis, lymphoplasmacytic infiltration, and follicular cell degeneration, representing rapidly progressive, destructive subtype of HT [27–31]. In the present study the patients with elevated serum IgG4 accounted for 32.5% of the group studied, and the percentage of male patients was 7.5% in the non-IgG4-HT and as much as 18.2% in the group with a high level of IgG4. Moreover, despite shorter duration of the disease, the patients with elevated IgG4 required higher doses of L-thyroxine; however, the observed difference was not significant. An ultrasound examination revealed that the signs of fibrosis were present in 27.0% of the IgG4-HT patients in comparison with 9.1% of the non-IgG4-HT group, suggesting more rapid and destructive form of HT.

In contrast to other studies [9, 28–32], the level of TPOAb was significantly lower in the patients with elevated IgG4 than the non-IgG4-HT individuals, whereas TRAb concentrations were similar in both groups with HT. This finding, together with the negative history of other autoimmune diseases in the IgG4-HT group, might suggest that the destructive variant of HT related to high serum IgG4 does not share the same autoimmune mechanisms with the “classic” form of Hashimoto's thyroiditis.

One of the crucial mechanisms potentially responsible for the induction and progression of HT is the apoptosis of both thyrocytes and T lymphocytes. It has been shown that Fas and Fas Ligand (FasL) proteins are expressed in normal thyrocytes, but their concentrations are insufficient to initiate apoptosis [33–37]. Changes in the apoptotic molecules expression on the T lymphocyte surface and thyroid follicular cells in patients with autoimmune thyroid diseases strongly suggest their vital role in the pathogenesis of HT [33–36]. The connection between FasL expression in thyrocytes and the prevention of autoimmune thyroiditis has also been proven [34–38]. Tumor necrosis factor- (TNF-) related apoptosis-inducing ligand (TRAIL) protein is another molecule possibly contributing to the autoimmune thyroid disease. Lymphocytes with TRAIL expression infiltrate the thyroid and in particular conditions may either induce TRAIL dependent apoptosis or “become the victim” of this process [38–40]. However, since then the role of TRAIL in HT pathogenesis remains unclear. Our analysis did not reveal any significant differences in circulating apoptotic markers and chemokines (CXCL9, 10, and 11) between the patients with elevated and low/normal-IgG4 level, as well as between the IgG4-HT group and the healthy controls. On the other hand, an increased level of TNF-*α* in the IgG4-TH group might suggest the pathogenic role for inflammatory processes more as IgG4-RD is regarded as a multiorgan fibroinflammatory condition. However, we should mention that the concentrations of other potentially proinflammatory chemokines did not differ markedly between the groups studied.

The main limitation of our study is the fact that the division into the IgG4-TH and non-IgG4-HT groups was made only on the basis of serum IgG4 level, without immunohistochemical analysis. However, we aimed to find out whether a single measurement of IgG4 concentration may help us to identify patients with a distinct form of thyroiditis.

In conclusion, our results suggest that the measurement of serum IgG4 allows for an early identification of patients with more rapid progressing and destructive form of HT, who require higher doses of L-thyroxine. A relatively low TPOAb level and the absence of coexisting autoimmune diseases may suggest a distinct pathomechanism of this type of thyroiditis. It should be mentioned that measuring serum IgG4 level may be a clinically useful marker for differentiating two distinct immunophenotypes of the disease. Furthermore, the identification of HT patients with high IgG4 levels might provide new therapeutic possibilities since there is good evidence that the use of oral glucocorticoids in patients with IgG4-RD results in a considerable clinical improvement.

## Figures and Tables

**Table 1:** Ultrasonographic and biochemical characteristics of the patients with non-IgG4-HT, IgG4-HT, and the control group.

	Non-IgG4-HT IgG4 < 135 IU/mL *n* = 40	IgG4-HT IgG4 > 135 IU/mL *n* = 13	*P*	Control group *n* = 28	*P* ^*^
Thyroid volume (mL)	12.1 (7.9–14.9)	13.0 (10.1–17.2)	ns	18.5 (12.5–19.5)	0.001
Thyroid nodules (*n*)	1.0 (1.0–2.0)	2.0 (1.0–2.0)	0.02	—	
TSH (IU/mL)	1.5 (0.98–1.9)	1.2 (0.95–2.1)	ns	1.1 (0.9–1.4)	
TPOAb (IU/L)	248.3 (40.4–413.3)	11.0 (4.6–294.9)	0.02	6.1 (4.9–10.2)	0.00001
TRAb (IU/L)	1.1 (0.8–1.3)	1.3 (0.8–1.7)	ns	0.3 (0.3–0.5)	0.00001
Levothyroxine dose (ug/day)	50.0 (0–81.3)	75.0 (50.0–88.0)	ns		

Data are shown as medians (interquartile range); differences between groups were tested by Mann-Whitney *U* test. *P*: between IgG4-HT and non-IgG4-HT; *P* ^*^: between IgG4-HT and control group.

**Table 2:** Body composition analysis in patients with non-IgG4-HT and IgG4-HT compared with the control group.

	Non-IgG4-HT IgG4 < 135 IU/mL *n* = 40	IgG4-HT IgG4 > 135 IU/mL *n* = 13	*P*	Control group *n* = 28	*P* ^*^
Age (years)	50.0 (33.5–58.0)	48.6 (38.0–56.0)	ns		
Body mass (kg)	74.1 (63.5–94.0)	73.6 (64.6–78.6)	ns	65.0 (60.0–74.1)	0.008
BMI (kg/m^2^)	27.5 (23.9–34.9)	26.0 (25.2–29.2)	ns	22.1 (20.7–27.1)	0.02
WHR	0.95 (0.89–1.01)	0.89 (0.83–0.99)	ns	0.84 (0.76–0.94)	0.01
TBW	34.5 (30.0–41.1)	35.8 (32.4–37.5)	ns	33.0 (31.0–36.9)	ns
FFA (kg)	47.2 (42.0–56.0)	45.8 (44.0–51.1)	ns	44.9 (41.1–49.0)	ns
Fat mass (kg)	29.1 (21.5–40.4)	26.4 (20.6–29.4)	ns	26.8 (20.6–37.1)	0.02
SMM (kg)	26.0 (22.6–31.1)	25.2 (24.1–28.0)	ns	24.5 (22.8–27.9)	ns
BMR (kcal/day)	1390.0 (1273.0–1581.0)	1360.5 (1322.0–1475.0)	ns	1342.9 (1282.0–1455.0)	ns

Data are shown as medians (interquartile range); WHR: waist/hip ratio, TBW: total body water, PBF: percentage of body fat, FFA: free fat mass, SMM: skeletal muscle mass, and BMR: basic metabolic rate; differences between groups were tested by Mann-Whitney *U* test; *P*: between IgG4-HT and non-IgG4-HT; *P* ^*^: between IgG4-HT and control group.

**Table 3:** Proinflammatory cytokines and apoptotic markers in the patients with non-IgG4-HT, IgG4-HT, and the control group.

	Non-IgG4-HT IgG4 < 135 IU/mL *n* = 40	IgG4-HT IgG4 > 135 IU/mL *n* = 13	*P*	Control group *n* = 28	*P* ^*^
IgG4 (IU/mL)	60.1 (25.3–75.9)	163.6 (131.5–208.7)	0.001	96.3 (42.7–112.2)	ns
TGF-*β*1 (pg/mL)	30361 (26019–32897)	33732 (29897–36800)	0.05	32293 (29017–38791)	ns
TNF-*α* (pg/mL)	5.7 (5.0–7.2)	6.1 (5.6–8.2)	ns	5.3 (4.1–6.4)	0.01
TRAIL (pg/mL)	109.2 (93.2–133.7)	110.3 (83.8–113.3)	ns	107.2 (87.3–129.6)	ns
Fas Ligand (pg/mL)	90.3 (76.6–104.9)	82.5 (74.2–130.2)	ns	87.7 (68.4–111.7)	ns
CXCL9 (pg/mL)	70.6 (52.7–95.7)	88.7 (69.1–116.4)	ns	62.7 (51.3–92.5)	ns
CXCL10 (pg/mL)	94.7 (79.9–120.7)	95.3 (77.4–130.2)	ns	96.0 (96.3–129.6)	ns
CXXL11 (pg/mL)	59.8 (24.7–77.2)	52.5 (24.7–63.4)	ns	56.2 (37.2–110.0)	ns

Data are shown as medians (interquartile range); differences between groups were tested by Mann-Whitney *U* test. *P*: between IgG4-HT and non-IgG4-HT; *P* ^*^: between IgG4-HT and control group.
